# Assessment of Donor‐Specific Human Leukocyte Antigen Antibodies Following Pediatric Liver Transplantation: Predictors, Protectors, and Clinical Relevance

**DOI:** 10.1111/ctr.70390

**Published:** 2025-11-21

**Authors:** Evelien Kanaan, Sinja Ohlsson, Simone Kathemann, Benas Prusinskas, Sofia Tsaka, Falko M. Heinemann, Andreas Heinold, Maren Schulze, Lars Pape, Elke Lainka

**Affiliations:** ^1^ Department of Pediatrics II University Children's Hospital Essen Germany; ^2^ Institute for Transfusion Medicine Transplantation Diagnostic, University Hospital Essen Germany; ^3^ Department of General Visceral‐ and Transplant Surgery University Hospital Essen Germany

**Keywords:** antibody‐mediated rejection, donor‐specific antibodies, human leukocyte antigen antibodies, pediatric liver transplantation

## Abstract

**Background:**

Following pediatric liver transplantation (pLT), the significance and management of donor‐specific antibodies (DSA) against human leukocyte antigen (HLA) remain undefined. The aim of this single‐center study was to investigate the occurrence of DSA, their clinical impact on predictors for and protectors against DSA.

**Patients and Methods:**

We compared anti‐HLA DSA (cutoff for mean fluorescence intensity (MFI) ≥ 1000), clinical and laboratory results and outcome in a routine (RG, *n* = 142, standard DSA testing) and a hepatopathy group (HG, *n* = 19, DSA testing following indeterminate hepatopathy) in 161 pLT patients, treated 2000–2021, retrospectively.

**Results:**

40% of RG and 32% of HG patients were DSA+ (39% of all patients, of which 13% with antibody‐mediated rejection [AMR]). Most frequent DSA subtypes were HLA‐DQ3, ‐DQ1, ‐DQ2 in RG and HLA‐DQ2, ‐DR15 in HG. MFI was higher for anti‐HLA II DSA (15 257 DSA+ vs. 5500 DSA−, *p *= 0.003), especially with AMR (21 000 DSA+ with AMR vs. 14 584 DSA+ without AMR, *p *= 0.042). Predictors for DSA included age at pLT, re‐pLT, and cystic fibrosis. Living donation and cold ischemia time <8 h appeared to offer protection. Graft survival was poorer with DSA (RG 78% DSA+ vs. 97% DSA−, *p *= 0.018, HG 67% DSA+ vs. 100% DSA−, *p *= 0.0007). Patient survival was 97% for the entire cohort.

**Conclusions:**

DSA were detectable in 39% and associated with AMR in 13% of children post‐pLT in addition to worse graft survival in all patients. Patient survival of 97% was not influenced. Potential DSA and predictors and protectors were identified. Therefore, DSA diagnostics are recommended after pLT.

**Abbreviations**
Ab: antibodies
AMR: antibody‐mediated rejection
CF: cystic fibrosis
CSA: cyclosporine A
DDLT: deceased donor liver transplant
DNA: deoxyribonucleic acid
DSA: donor‐specific antibodies
HG: hepatopathy group
HLA: human leukocyte antigen
LDLT: living donor liver transplant
MFI: mean fluorescence intensity
MPA: mycophenolic acid
PCR‐SSO: polymerase chain reaction with sequence‐specific oligonucleotide probes
pLT: pediatric liver transplantation
RG: routine group
TAC: tacrolimus
TCMR: T‐cell‐mediated rejection

## 1 Introduction

In recent years, donor‐specific antibodies (DSA) against human leukocyte antigen (HLA) after pediatric liver transplantation (pLT) has attracted increasing scientific attention. Nevertheless, the number and significance of related studies, especially concerning pediatric patients, are limited. Most studies examine adult patients, and although one meta‐analysis demonstrated a higher prevalence of DSA in pLT recipients than in adults [1], only patient populations from individual centers are analyzed and reported. The available literature underlines the need for more data, as DSA have been shown to be associated with liver graft fibrosis progression, rejection, and detrimental outcomes [2, 3].

The liver is known to be the immunologically most “tolerogenic” transplant organ. This is one reason why fewer rejections occur and smaller dosages of immunosuppressants are needed compared to other organ transplantations [4]. The liver parenchyma is protected by various immunological mechanisms, the immense capillary surface and good regenerative capacity [5]. Hence, impact of DSA on the transplant organ can be reduced, but not prevented entirely. Until recently, HLA‐compatibility and DSA were not considered relevant in pLT. Antibody‐mediated rejection (AMR) has increasingly become the object of investigation and discussion. Meanwhile, operational tolerance by complete weaning from immunosuppressants is also increasingly under discussion, as it is an aspirational goal in future patient care. Feng et al. showed that the presence of DSAs does not necessarily indicate unsuccessful operational tolerance in pLT [6].

In AMR, antibodies (Ab) of the recipient against the AB0 blood group antigens in AB0‐incompatible transplants or against the organ donor's HLA antigens (DSA) cause damage to the donor organ [7]. Ab can be performed prior to pLT, for example, through previous contact with foreign antigens via blood transfusions, or develop de novo after contact with the donor organ [8]. Endothelial cells and the arterial capillary plexus around the bile ducts appear to be particularly affected by AMR, resulting in ischemic cholangiopathies [9, 10]. It is assumed that acute AMR, especially after prior existing DSA, often progresses rapidly to T‐cell mediated rejection (TCMR) [11, 12, 13]. Chronic rejection rather leads to slowly progressive symptoms of graft dysfunction. Persistent DSA levels and poorly adjusted immunosuppression seem to play a role here [14].

Although AMR and DSA have been subject of scientific research more frequently, there are still many unanswered questions, especially around the lack of recommendations regarding practical clinical management. The intention of this study was to investigate the occurrence of DSA, to obtain more information on potential predictors for their development after pLT, as well as possible protective factors as indicators for potential measures to prevent AMR. By analyzing survival rates, our aim was to investigate the impact of DSA, as their clinical relevance in pLT is still a point of controversy.

## 2 Patients and Methods

### 2.1 Patients

**FIGURE 1:**
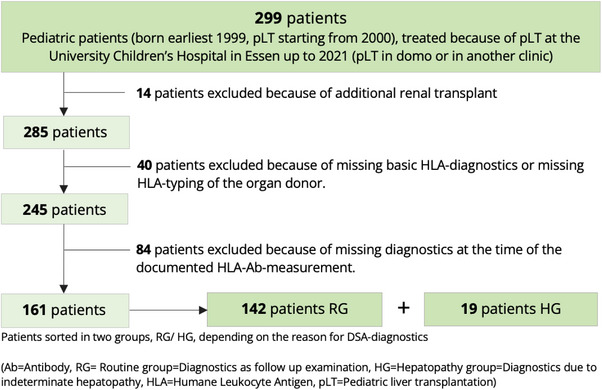
Patient exclusion criteria.

This retrospective monocentric study included 161 children after pLT who were treated between 2000 and 2021. Inclusion criterion was an available HLA Ab measurement. Patients with both kidney and liver transplantation were excluded as well as patients with incomplete diagnostics (e.g., missing HLA typing of the organ donor) (Figure 1).

Two groups were formed: (1) Routine group (RG, *n* = 142, DSA screening performed as part of routine follow‐up after pLT), (2) Hepatopathy group (HG, *n* = 19, DSA diagnostics performed due to indeterminate hepatopathy). Hepatopathy was defined as transaminases elevated over twice the upper value, fibrosis ≥F2 or a definite liver disease diagnosis. Within the two patient groups (RG, HG), subgroups were formed regarding the presence of DSA (DSA+, DSA−). General patient characteristics and key information regarding the pLT (e.g. deceased donor, ischemia time) were documented as well as various clinical and laboratory results including immunosuppressive medication and liver enzymes. Additionally, sonography, liver elastography and results of the liver biopsy were collected (Table 1). These findings were documented for two points in time – 1. surrounding the date of the pLT and 2. at the time of a later HLA‐Ab measurement (e.g., at routine follow‐up 1 year after pLT). The setting of the study thus is one timepoint cross sectional. The study was approved by the ethics committee of the faculty of medicine of the University Duisburg‐Essen (18‐8231‐BO).

### 2.2 HLA Typing and Testing for Antibodies

Patient deoxyribonucleic acid (DNA) was isolated from peripheral blood samples. DNA was extracted using spin columns (Qiagen, Hilden, Germany) or by an automated system, using magnetic beads (Chemagen/Perkin Elmer, Baesweiler, Germany). HLA typing of the recipients and living donors was performed for the HLA‐A, B, C, DRB1 and DQA1/DQB1 loci by PCR‐SSO (polymerase chain reaction with sequence‐specific oligonucleotide probes) in single‐field resolution and a Luminex system (LABType by One Lambda/Thermo Fisher, Canoga Park, CA, USA). Deceased donors were typed according the Eurotransplant allocation procedures [15]. Patient post transplantation serum samples were tested for IgG Ab against HLA class I and II using Luminex‐technology (LABScreen‐Mixed Beads, One Lambda/Thermo Fisher, Canoga Park, CA, USA) according to the manufacturer's instructions and as described in detail previously [16, 17]. If results for HLA class I or II Ab were positive, the Luminex Single Antigen Beads LABScreen assay was used, provided by the same manufacturer, to confirm if HLA Ab were donor specific. Mean fluorescence intensity (MFI) of ≥1.000 was considered positive regarding evidence for DSA. The focus of this study was exclusively on DSA after liver transplantation. HLA antibody analyses for preformed DSA had not been performed regularly and pre‐TX antibody data was not included.

### 2.3 Data Collection and Statistical Analysis

All data were collected retrospectively by reviewing the patient files in electronic (CGM MEDICO, Koblenz, Germany), paper‐based documentation systems and the pLT database of the University Children's Hospital Essen. Statistical analysis was carried out using GraphPad Prism, version 9.3.1 (1994–2023 GraphPad Software, LLC) and Microsoft Excel for Mac version 16, 2021 (Microsoft Corporation, Washington, USA). Data collected from both groups were analyzed descriptively. The *t*‐test, Mann–Whitney *U* test or Chi‐square test were used for comparisons between groups. The Pearson correlation coefficient was calculated to identify possible correlations. To investigate patient and graft survival Kaplan–Meier curves were created, combined with the log‐rank test to analyze significant differences. If one patient showed DSA against multiple HLA‐specificities, the highest found MFI strength was used for calculations, not the sum or mean of all values. Significance was always assumed at a *p* value < 0.05.

## 3 Results

### 3.1 Patient Cohort

One hundred and sixty‐one children (51% male, 49% female) were a median of 12 months old (range 3–205 months) at pLT (Table 1). The main indication for pLT was biliary atresia 50%, followed by progressive familial intrahepatic cholestasis types I–III 11%, acute liver failure with unknown cause 7%, several diseases of the biliary tract (e.g., primary sclerosing cholangitis, choledochal cyst) 7%, metabolic diseases 5%, Alagille‐syndrome 4%, hepatoblastoma 3%, cystic fibrosis (CF) 4%, and others.

**TABLE 1:** Patient cohort.

	Routine group	Hepatopathy group	Complete cohort
Number of patients (*n*)	142	19	161
Sex	73 male (51%) 69 female (49%)	9 male (47%) 10 female (53%)	82 male (51%) 79 female (49%)
**At the time of pLT**			
Age at first pLT (months)	42.4 ± 55.7 Median 13 Range 3–204	54.8 ± 71.65 Median 14 Range 5–205	43.9 ± 57.7 Median 12 Range 3–205
Mean height (cm)	86.3 ± 32.1 Median 70.5 Range 49–183	94.9 ± 39.7 Median 76.5 Range 58–173	87.3 ± 33.1 Median 71 Range 49–183
Mean weight (kg)	15 ± 17.8 Median 8 Range 4–119	21.2 ± 25.1 Median 10.2 Range 4.5–86	15.9 ± 18.7 Median 8 Range 4.5—119
Body surface area (m^2^)	0.62 ± 0.48 Median 0.4 Range 0.24–3.4	0.7 ± 0.56 Median 0.49 Range 0.27–1.99	0.63 ± 0.49 Median 0.4 Range 0.24–3.4
Death following pLT (*n*)	2/142 (1%)	2/19 (11%)	4/161 (3%)
Cold ischemia time <8 h (*n*)	77/129 (60%)	11/17 (65%)	88/146 (60%)
Warm ischemia time <45 min (*n*)	93/126 (74%)	15/18 (83%)	108/144 (75%)
AB0‐BG‐incompatibility	6/142 (4.2%)	4 /19 (21.1%)	10/161 (6.2%)
Full organ (*n*)	53/141 (38%)	8 /19 (42%)	61/160 (38%)
Deceased donor organ (*n*)	93/141 (66%)	12/19 (63%)	105/160 (66%)
Re–pLT (*n*)	15/142 (11%)	2 /19 (11%)	17/161 (11%)
**Results at the time of the HLA Ab measurement**			
Time since pLT (months)	64.7 ± 61.5 Median 36 Range 2–205	47 ± 55.6 Median 24 Range 0–183	63 ± 61 Median 35 Range 0–205
Hepatopathy at the time (*n*)	22/142 (16%)	19/19 (100%)	4/142 (28%)
Hepatopathy at any time, up to 2021 (*n*)	79/140 (56%)	19/19 (100%)	95/159 (60%)
Binding HLA Ab (*n*)	84/142 (59%)	17/19 (90%)	101/161 (63%)
HLA‐class I Ab (MFI >1000, *n*)	25/142 (18%)	10/19 (53%)	35/161 (22%)
HLA‐class II Ab (MFI >1000, *n*)	64/142 (45%)	10/19 (53%)	74/161 (46%)
DSA+ in this examination (*n*)	57/142 (40%)	6/19 (32%)	63/161 (39%)
DSA+ at any time, up to 2021 (*n*)	67/142 (47%)	8/19 (42%)	75/161 (47%)
GGT (U/l)	31.6 ± 64.9 Median 15 Range 4–498	262.1 ± 247.3 Median 212 Range 7–866	58.8 ± 127.1 Median 16 Range 4–866
GOT (U/l)	39.6 ± 40.3 Median 31 Range 8–393	163.2 ± 166.3 Median 108 Range 35–663	54.2 ± 78.4 Median 33 Range 8–663
GPT (U/l)	35 ± 34.2 Median 25 Range 7–215	227.8 ± 253.8 Median 132 Range 24–1049	57.8 ± 110.3 Median 27 Range 7–1049
Total bilirubin (mg/dL)	0.8 ± 2.2 Median 0.5 Range 0–25.1	4 ± 7.4 Median 0.6 Range 0.2–26.2	1.2 ± 3.4 Median 0.5 Range 0–26.2
Direct bilirubin (mg/dL)	0.4 ± 1.6 Median 0.2 Range 0–18.6	3.3 ± 6.4 Median 0.3 Range 0.1–21.6	0.7 ± 2.8 Median 0.2 Range 0–21.6
Quick value (%)	85.2 ± 13.5 Median 86 Range 51–119	84.3 ± 19.6 Median 83 Range 38–112	85.1 ± 14.3 Median 86 Range 38–119
Albumin (g/dL)	4.4 ± 0.4 Median 4.5 Range 2.3‐5.4	4 ± 0.6 Median 4.1 Range 2.1–4.7	4.4 ± 0.4 Median 4.4 Range 2.1–5.4
Creatinine (µmol/L)	51.8 ± 20.5 Median 51 Range 17–136	54.9 ± 43.1 Median 49.5 Range 17–217	52.2 ± 23.9 Median 50.5 Range 17–217
Immunoglobulin G (g/L)	10 ± 3.6 Median 9.9 Range 1.4–19.8	14 ± 7.3 Median 12.4 Range 5.7–30.2	10.3 ± 4.2 Median 10 Range 1.4–30.2
**Imaging procedures**			
Valid liver elastography result (*n*)	70/142 (49%)	9/19 (47%)	79/161 (49%)
Pathologic elastograpy result (kPa)	>5 = 36/70 (51%) >7.3 = 10/70 (14%) >9.6 = 3/70 (4%) Mean 5.7 ± 2.3 Median 5 Range 0.5–17.6	>5 = 8/9 (89%) >7.3 = 4/9 (44%) >9.6 = 2/9 (22%) Mean 9.1 ± 7.7 Median 6.6 Range 0.6–27.4	>5 = 44/79 (56%) >7.3 = 14/79 (18%) >9.6 = 5/79 (6%) Mean 6.1 ± 3.5 Median 5.2 Range 0.5–27.4
Liver biopsy performed (*n*)	35/142 (25%)	19/19 (100%)	54/161 (34%)
Fibrosis detected (F ≥ 1) (*n*) Fibrosis scoring	22/35 (63%) Mean 1.9 ± 0.8 Median 2 Range 1‐4	18/19 (95%) Mean 1.9 ± 1.1 Median 1.5 Range 1–4	40/54 (74%) Mean 1.9 ± 0.9 Median 1.8 Range 1‐4
Fatty degeneration (*n*)	5/35 (14%)	2/19 (11%)	7/54 (13%)
Rejection (*n*)	3/35 (9%)	5/19 (27%)	5/54 (9%)
Inflammation detected (Score ≥ 1) (*n*) Inflammation score	16/35 (46%) Mean 2.2 ± 0.8 Median 2 Range 1–3	13/19 (68%) Mean 1.6 ± 0.8 Median 1 Range 1–3	29/54 (54%) Mean 1.9 ± 0.8 Median 2 Range 1–3
ITBL	3/35 (9%)	4/19 (21%)	7/54 (13%)

*Notes:* Varying main unit due to missing result in some cases. Metric data shown as: Mean ± standard deviation, median, range (minimum‐maximum).

Abbreviations: Ab, antibody; AB0‐BG, AB0‐blood group; DSA, donor‐specific HLA‐antibodies; GGT, gamma‐glutamyl‐transferase; GOT, glutamate‐oxalacetat‐transaminase; GPT, glutamat‐pyruvat‐transaminase; HLA, humane leukocyte antigen; ITBL, ischemic type biliary lesions; MFI, mean fluorescence intensity; pLT, pediatric liver transplantation.

### 3.2 Survival

**FIGURE 2:**
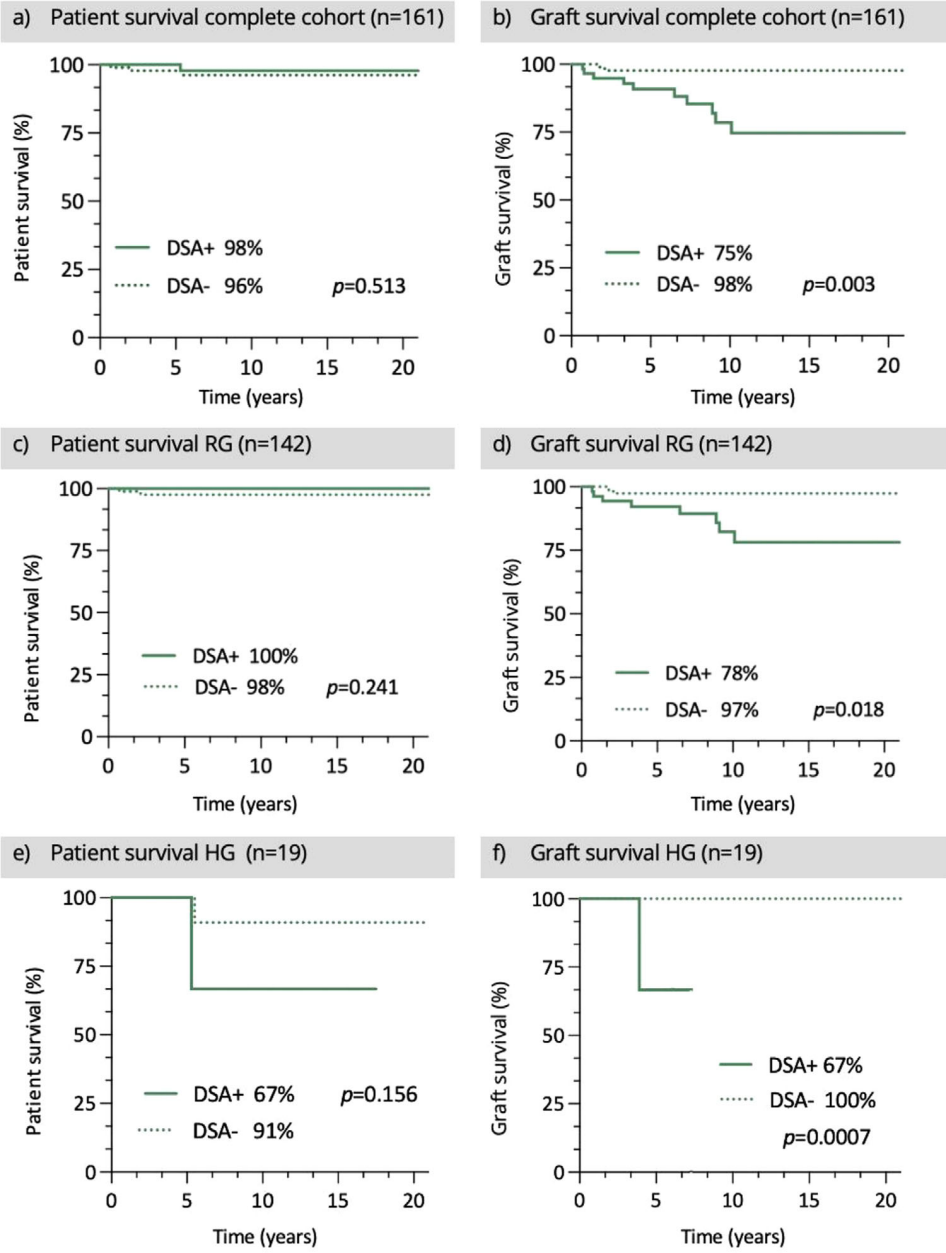
Patient and graft survival, comparison DSA+ versus DSA−.

Overall graft survival was significantly higher in the DSA− than in the DSA+ subgroup, *p* = 0.003. Graft survival was lower when DSA+ were present (RG 78% DSA+ vs. 97% DSA−, *p* = 0.018, HG 67% DSA+ vs. 100% DSA−, *p *= 0.0007). Patient survival showed no significant difference, RG *p *= 0.241, HG *p *= 0.156. Patient survival of the entire cohort (*n* = 161) was higher for DSA+ than for DSA−, but not significantly, *p *= 0.513 (Figure 2). Four children died over time: two children from the HG (1 DSA−, 1 DSA+), two children from the RG (both DSA−).

### 3.3 Routine Group (RG)

#### 3.3.1 DSA Screening

One hundred and forty‐two patients had been tested for DSA in a routine follow‐up setting. DSA+ were found in 40%, 57 patients; 60%, 85 patients were DSA−. The most frequent DSA specificity was HLA‐DQ3, 33% [19/57], including the splits ‐DQ7, ‐DQ8, ‐DQ9. Among these, Ab against HLA‐DQ7 were the most frequent (*n* = 12). Next were HLA‐DQ1 (including the splits HLA‐DQ5 and ‐DQ6) 32% [18/57] and HLA‐DQ2 16% [9/57]. 98% [56/57] of all DSA+ had HLA class II Ab, only 28% [16/57] of which showed HLA class I Ab.

MFI was ≥5000 in 86% [48/56] of the cases with donor‐specific HLA class II Ab, and in 66% [37/56] MFI was ≥10 000. For donor‐specific HLA class II Ab, the mean MFI was 15 257, significantly higher than without donor specificity 5500, *p *= 0.005. For HLA class I Ab, the difference in MFI between DSA+ (mean MFI 6013) and DSA− (mean MFI 4900) was not significant.

**FIGURE 3:**
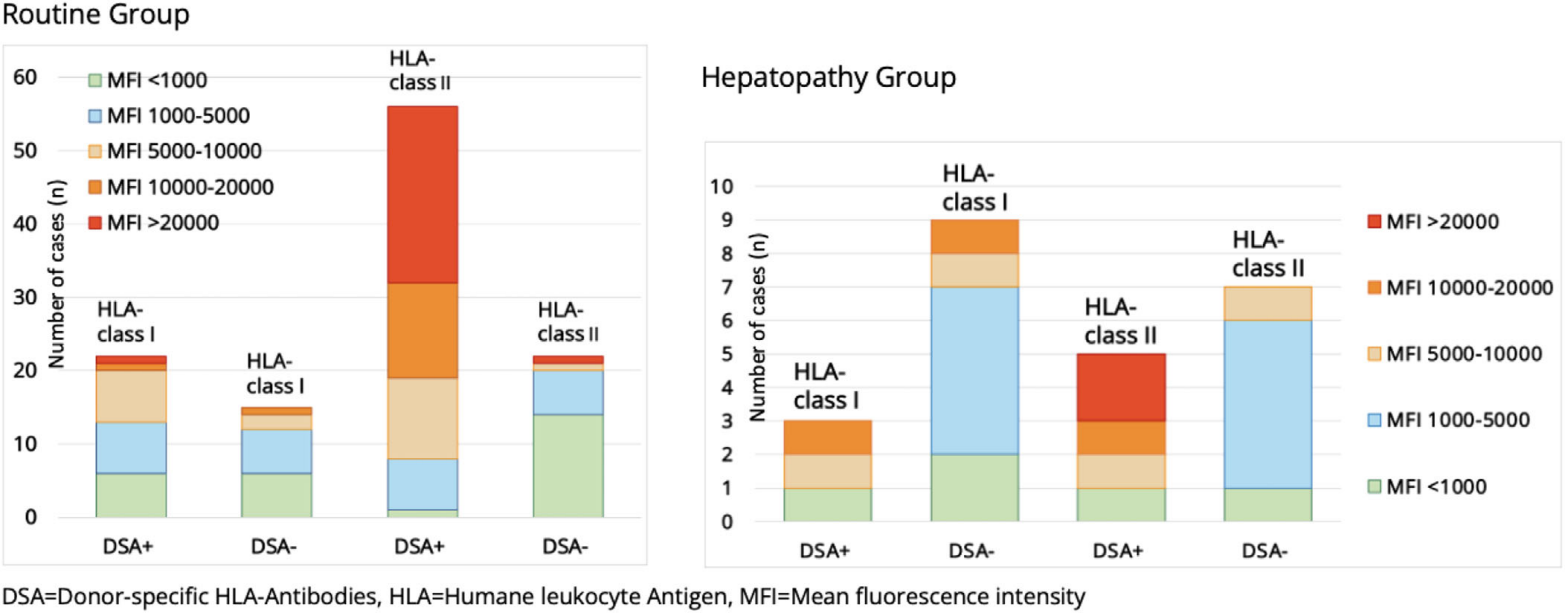
Strength of MFI for antibodies against HLA molecules depending on donor specificity.

Thirty‐two patients showed DSA+ and MFI ≥ 15 000.19% [6/32] patients suffered from hepatopathy.67% [4/6] cases were diagnosed with AMR. DSA+ and MFI < 15 000 were detected in 25 patients.16% [4/25] children suffered from hepatopathy, of which one case was an AMR (25%, [1/4]). Even though the proportion of those with AMR was higher for MFI ≥ 15 000, this difference was not significant. Conversely, 79% of DSA+ and MFI ≥ 5000 showed no hepatopathy, 81% of DSA+ and MFI ≥ 15 000 and 83% of DSA+ and MFI ≥20 000 had no clinical problems (Figure 3).

16% [22/142] of all RG patients, 39% [22/57] of all DSA+ showed hepatopathy, the most frequent underlying diagnosis being DSA‐related AMR 23% [5/22], fibrosis 23% [5/22] and cholangitis 18% [4/22], followed by other biliary tract related problems and blood vessel complications both 9% [2/22].

#### 3.3.2 Basic Characteristics

Patients with DSA+ were significantly older (DSA+ 52.6 ± 61.5 months vs. DSA− 35.5 ± 59.6 months, *p *= 0.004) and taller at the time of pLT (DSA+ 95.4 ± 35 cm vs. DSA− with 84.6 ± 30.8 cm, *p *= 0.03). Age and height correlated, as assumed (*r* = 0.933). In DSA+ patients, deceased donor liver transplants (DDLT) were significantly more common (DSA+ 80% [45/56] vs. DSA− 56% [48/85], *p *= 0.003). If cold ischemia time had been less than 8 h, DSA were significantly less frequently detected (DSA+ 49% [25/51] vs. DSA− 67% [52/78], *p *= 0.028). Re‐pLT was more common in DSA+ than in DSA− (DSA+ 19% [11/57] vs. DSA− 5% [4/85], *p *= 0.004). CF was the only underlying disease with a significantly higher proportion of affected patients among DSA+ (DSA+ 9% [5/57] vs. DSA− 1% [1/85], *p *= 0.025). The time from pLT to documented time of DSA testing was significantly higher for DSA+ (DSA+ 77 ± 61 months vs. 52 ± 56 months, *p *= 0.006).

#### 3.3.3 Immunosuppressive Medication

The proportion of patients taking cyclosporine A (CSA) at the time of the DSA screening was significantly higher in DSA+ cases than in those with DSA− (DSA+ 19% [11/57] vs. DSA− 7% [6/85], *p *= 0.028). No significant differences were detected for the use of tacrolimus (TAC), predniso(lo)ne or mycophenolic acid (MPA).

### 3.4 Hepatopathy Group (HG)

#### 3.4.1 DSA Diagnostics

DSA+ were detected in 32% [6/19] patients and DSA− in 68% [13/19]. If DSA+ were found, they were most frequently directed against HLA‐DQ2 16% [3/19], followed by HLA‐DR15 11% [2/19]. The average time between pLT and HLA Ab examination was 47 ± 55 months. The proportions of Ab against HLA class I and II did not differ significantly between DSA+ and DSA−. HLA−Ab against class II showed a significantly higher MFI with donor specificity than without (DSA+ 18 700 vs. DSA− 3567, *p *= 0.019) (Figure 3). 16% [3/19] patients suffered from hepatopathy with underlying DSA‐related AMR. The following most frequent reasons for hepatopathy were biliary tract complications 21% [4/19] (e.g., stenosis, mismatch), HLA‐unrelated rejection, Epstein Barr virus infection and autoimmune hepatitis, each 11% [2/19].

#### 3.4.2 Basic Characteristics

Similar to the RG cohort, patients were significantly older at pLT, when DSA were detected (DSA+ 101 ± 89 months vs. DSA− 33 ± 53 months, *p *= 0.028). DSA+ were more frequent in patients after re‐transplantation (DSA+ 33% [2/6] vs. DSA− [0/13] *p *= 0.028). No other significant differences were found for basic characteristics.

#### 3.4.3 Immunosuppressive Medication

Predniso(lo)ne was taken significantly more frequently at the time of the DSA diagnostics, if DSA+ were detected (DSA+ 100% [6/6] vs. DSA− 54% [7/13], *p *= 0.044). For MPA, similar results were found (DSA+ 100% [6/6] vs. DSA− 23% [3/13], *p *= 0.002). There was no significant difference in the use of CSA and TAC.

## 4 Discussion

Due to the scarcity of liver donors and the significantly worse outcomes associated with re‐transplantation, there is an unmet need to prolong graft survival by diagnosing rejections promptly and improving patient management after pLT. The literature on DSA is not only sparse, but often also controversial, as in our study.

In our cohort, DSA+ were detected in 39% [63/161] patients. This is close to the average prevalence among pediatric patients of 41% reported in a meta‐analysis from 2016 [18].

Similar to other studies, DSA+ were significantly more often specified against HLA class II antigens than against HLA class I antigens. The predominant expression of HLA I molecules in the liver may lead to increased formation of Abs against these molecules but also appears to result in increased absorption of Abs in the transplant organ. Presumably, this reduces the concentration of DSA against HLA class I in peripheral blood. Intrahepatic soluble HLA class I molecules, forming immune complexes with class I DSA, which are then eliminated by Kupffer cells, play an important role here [19, 20]. This theory is also supported by the observation that the liver appears to have a protective effect on the transplanted kidney in simultaneous liver‐kidney transplantations from the same donor, at least regarding preformed DSA. In some such cases (in adults), complete disappearance of HLA class I DSA, and even DSA against both HLA classes, has been demonstrated after transplantation [21, 22].

A significantly higher mean MFI was found for HLA class II Ab, when donor specificity was present. Patients with AMR showed MFI values between 14 000 and 28 600, clearly above the cutoff of 1000. The comparison of MFI values between DSA+ with AMR present against those in DSA+ without AMR (for RG + HG, *n* = 161) showed significantly higher MFI values for the group with AMR (DSA+ and AMR mean MFI 21 000 ± 5224, DSA+ without AMR mean MFI 14 584 ± 8465, *p *= 0.0427). At the same time, around 77% of DSA+ with an MFI ≥ 5000 had no hepatopathy, and 79% of DSA+ with an MFI of ≥20 000 also showed no clinical problems. Thus, these results indicate AMR being unlikely at very low MFI, and the combination of hepatopathy and DSA+ with high MFI (≥15 000) should possibly lead to considering AMR as a diagnosis. At the same time, however, high MFI values alone do not seem to be a good marker for adverse clinical issues. On the one hand, more clinical problems such as fibrosis and higher mortality are reported to be associated with higher MFI values [23, 24], whilst on the other hand, association of low MFI values (500–4000) with clinical complications such as TCMR and AMR [25, 26] have been reported, as well as a clinical course completely independent from the MFI level [27, 28].

Another relevant factor is the HLA subtype. The fact that primarily DSA+ against the HLA subtypes ‐DR and ‐DQ were detected is consistent with the results of other pediatric studies [22, 28]. Melere et al. reported an association of HLA‐DQ DSA with a higher risk of rejection [29]. This is supported by our results, as rejection episodes occurred more often in patients with HLA‐DQ DSA than in those without (DQ+ 52% [22/42] vs. DQ− 7% [1/15], *p *= 0.002). All five patients with AMR were positive for HLA‐DQ DSA in the RG cohort and 2/3 AMR patients in the HG cohort. The frequency of the HLA alleles differs between populations (and thus among the donors), so the dominance of certain subtypes might not be expected in all populations. In addition, HLA‐DQ is particularly rarely expressed in the liver, leading to less absorption of the corresponding liver antibodies, which could be the reason for the predominant detection of HLA‐DQ Ab [20].

### 4.1 Potential Predictors

**Patient Age and Height at pLT**

Within the RG, patients with DSA+ were older and taller at the time of pLT than those without DSA−. It can be assumed that the patient's age played the decisive role here, considering that immunology changes over the course of the first years of life and body measurements depend on age. Other studies described that children with DSA+ were younger than those without DSA− [29]. In alignment with our results, Goto et al. found a higher age at DSA+ [23].

**Cystic Fibrosis**

In our study, CF was the only transplant indication associated with significantly more cases of DSA+. Theoretically, an increased probability of alloimmunization can be suspected due to the pronounced inflammatory processes already present in these patients before transplantation. After lung transplantation, CF was postulated as a risk factor for the development of DSA and AMR [31]. The adjustment of treatment is often more complex in patients with CF due to limited gastrointestinal absorption and increased renal elimination [32, 33].

**Re‐Transplantation**

Our results and those reported previously have identified re‐pLT as a risk factor for the development of DSA+ (adult patients) [34]. This is most likely related to the immunological processes stimulated by repeated transplants whereby the recipient's immune system has increased contact with foreign HLA antigens.

### 4.2 Potential protectors

**Cold Ischemia Time < 8 h**

In the RG, fewer DSA+ were detected if cold ischemia time during pLT had been <8 h. Other studies found no significant difference in ischemia time when comparing DSA+ and DSA− [23, 35, 36]. It is known that ischemia time is a significant risk factor for the stimulation of immunological processes in the transplant organ. Intrahepatic sinusoidal endothelial cells are particularly sensitive to cold ischemia [37]. The ischemia period creates an overall pro‐inflammatory situation, which increases the expression of both HLA class I and HLA class II antigens on hepatic and biliary endothelial cells and hepatocytes, potentially resulting in increased development of DSA [9]. Donor dendritic cells are increasingly transported from the transplant organ to the lymph nodes of the recipient, which in turn favors a corresponding immune response [10].

**Living Donation**

To date, there are discrepant findings on the role of the origin of transplant (DDLT vs. living donor liver transplant [LDLT]) on DSA development. DSA+ was found to be more frequent after DDLT than after LDLT in our RG. The fact that the immunological processes surrounding transplantation and the outcome differs between living and deceased donation have already been highlighted [38]. Besides the prolonged cold ischemia time, it is assumed that brain death, which precedes most DDLT, is one of the underlying reasons for this. Both factors lead to increased inflammation in the donor organ [39, 40]. DSA+ were detected less frequently in pediatric patients after living maternal donation. In addition to the higher probability for HLA compatibility due to the close relationship, maternal microchimerism, that is, the retention of maternal cells, including immune cells, in the child's organism, has been discussed [41]. Immunological processes surrounding DDLT thus may increase the risk of developing DSA.

**Immunosuppressive Medication**

It is hypothesized that suboptimal drug adjustment and poor adherence to therapy, leading to inadequate TAC target levels, favors the development of DSA+ and secondarily leads to therapy intensification [35, 42]. Finally, inadequate immunosuppression is known to increase the risk of alloimmune reactions, including the formation of DSA+ and the occurrence of rejection reactions [27, 43].

**Patient and Graft Survival**

The patient survival of our entire cohort was 97%, the time between pLT and the time point under consideration being on average about 5 years. Thus, patient survival is comparable to the result of 97.5% for patients treated in Hannover, Germany, 2014–2020 [44]. The graft survival for the whole cohort was 87%, which is closely in line with data from other studies, which vary from 72% to 93% at 5 years after pLT [45, 46]. It was significantly higher for DSA− than for DSA+ in both groups.

Thus, the present results therefore suggest that the detection of DSA+ represents a risk factor for graft survival probability. Schluckebier and colleagues describe an association between rejection reactions and DSA+ in children but found no change in graft survival [47]. In contrast, other authors have reported reduced graft survival in DSA+ in children [27] and adults [48, 49]. Kaneku et al. found worse values for both graft and patient survival in adults with DSA+ detection [20].

### 4.3 Limitations

The retrospective design and the small pediatric cohort limit this monocentric study. The patients are heterogeneous in terms of their age at pLT and the time between pLT and DSA screening. Missing HLA‐typing of the donor (e.g., after pLT in other countries) was frequently an exclusion criterion. Only a single HLA Ab examination and its ensuing results were documented rather than those over a long‐term course. Longitudinal HLA Ab tests may be used as noninvasive prognostic biomarkers for rejection in addition to liver enzymes and invasive liver biopsy. Modern precision post‐transplant care mandates comprehensive analysis of serological, histological, and functional biomarkers for optimal patient and graft management.

## 5 Conclusions

DSA+ were detectable in 39% of children after pLT and associated with AMR in 13%. DSA+ were associated with poorer graft survival. Predictors for DSA included age at pLT, re‐pLT, and CF. Living donation and cold ischemia time <8 h were found to have a protective role. Routine DSA diagnostics are therefore recommended, particularly in cases of hepatopathy.

## Author contributions

**Evelien Kanaan:** study design, investigation, data preparation, statistical analysis, writing. **Elke Lainka:** investigation, writing, review, editing, supervision, conceptualization. **Simone Kathemann:** investigation, drafting of manuscript, critical review. **Lars Pape:** critical review. **Sinja Ohlsson:** investigation, statistical analyses. **Maren Schulze:** critical review. **Falko M. Heinemann:** investigation, data preparation. **Andreas Heinold:** investigation, data preparation. **Benas Prusinskas:** investigation, critical review. **Sofia Tsaka:** investigation, critical review. All authors have read and agreed to the published version of the manuscript.

## Funding

The author has nothing to report.

## Conflicts of Interest

There are no conflicts of interest. All authors declare that they have no known competing financial and not financial interests that could have appeared to influence the work in this manuscript.

## Data Availability

The data that support the findings of this study are available on request from the corresponding author. The data are not publicly available due to privacy or ethical restrictions.
